# Are narcissists more attracted to people in relationships than to people not in relationships?

**DOI:** 10.1371/journal.pone.0194106

**Published:** 2018-03-27

**Authors:** Amy B. Brunell, Joshua Robison, Nicholas P. Deems, Bradley M. Okdie

**Affiliations:** Department of Psychology, The Ohio State University, Columbus, OH, United States of America; Brown University, UNITED STATES

## Abstract

Does grandiose narcissism predict greater attraction for others in relationships? We examined this question by replicating previous work implicating grandiose narcissists as mate poachers (Studies 1 and 2). We then used an experimental paradigm (Studies 3 and 4) to assess the extent to which grandiose narcissists indicate a greater interest in someone who is already in a relationship compared to someone who is single. Results suggest that although grandiose narcissism related to reports of more frequent mate poaching attempts, grandiose narcissists did not appear to be more interested in taking someone away from an existing relationship. Instead, participants took their own relationship status into consideration (rather than the relationship status of a target) when evaluating their interest in a target for a short-term fling or a long-term relationship. Thus, although grandiose narcissists report more frequent mate poaching attempts, they do not appear to be more interested in people in relationships compared to those who are single.

## Introduction

People often perceive individuals in relationships as more desirable [1], yet not everyone attempts to steal someone else’s significant other. In this paper, we examine the extent to which those who are higher in grandiose narcissism report greater attraction for people who are already in relationships. For brevity, we use the term “grandiose narcissists” when discussing those who score higher on trait grandiose narcissism.

Grandiose narcissism is marked by selfishness, arrogance, inflated self-views, and high extraversion paired with low neuroticism and agreeableness [2, 3]. Grandiose narcissists believe they are special and unique, entitling them to more than others [4]. They take advantage of others [5], experience less guilt for their transgressions [6], and are less moral in their reasoning about their everyday behavior that could potentially be harmful to others [7]. Despite these negative qualities, grandiose narcissists are charming and socially skilled [8], aiding their ability to attain positions of leadership and power [9]. Grandiose narcissists like to brag and show off [10]. They view themselves as powerful [11] and one way they maintain power in relationships is to keep their partners guessing about their interest and commitment [12, 13].

Grandiose narcissists report greater endorsement of casual, uncommitted sex, more lifetime sexual partners, and a greater desire for short-term mates [14]. The desire for power and influence links grandiose narcissism and sexual attitudes and behavior [15]; this includes sexual coercion among women and sexual aggression among men [16]. Grandiose narcissists also self-report more frequent mate poaching [17, 18], defined as behaviors that are enacted with the intention of attracting someone who is already in a romantic relationship for a sexual encounter [19, 20].

Grandiose narcissists prefer to engage in short-term mating strategies rather than long-term committed relationships [14, 15, 21]. Grandiose narcissists are perceived by others as sexy [22], which likely aids them in attracting short-term mates and possibly exciting sexual desire in others [23]. Likewise, people who engage in attractiveness self-enhancement are able to attain more (short-term) sexual partners [24], and grandiose narcissists are known to inflate their own ratings of attractiveness [25]. This might lead them to pursue short-term mates beyond those who are not actually available to them or “out of their league” [26].

The notion that grandiose narcissists might be attracted to short-term mates who are not actually available might explain their increased self-reports of mate poaching attempts. Mate poaching is fairly common; in one study 50% of people reported having attempted to poach a mate at some point in their lives [19]. Poaching a mate can be for a short-term “hook up,” a long-term, ongoing affair, or a new permanent relationship [27].

Grandiose narcissists tend to report mate poaching [17]—especially for the short-term [18]. However, a question remains as to whether they find people in relationships more worthy of pursuing than people who are single. Widman and McNulty [28] reason that grandiose narcissistic tendencies might be activated across situations on average (such as the self-report of lifetime prevalence of sexual behavior), but not be activated when placed in a specific situation. Thus, it is possible that grandiose narcissists disclose a history of engaging in mate poaching, but do not necessarily find those in relationships to be better mates. They might not be aware that they find targets in relationships as more alluring than single targets. We examined this possibility in four studies.

Studies 1 and 2 were set up to replicate and extend previous research on grandiose narcissism and mate poaching by examining if grandiose narcissism predicted self-reported mate poaching. However, because grandiose narcissists come across as sociable and charming [8], it is possible that their social charm is implicated in mate poaching rather than grandiose narcissism per se. For example, extraversion has been linked to greater sexual promiscuity [20], such as having more one-night stands [29]. Therefore, we control for the Big Five personality factors as they are linked to human sexual behaviors [30]. Controlling for Big Five personality was done in only one study examining grandiose narcissism and self-reports of mate poaching, and this study examined short-term poaches only [18]. Thus, our studies extend research on grandiose narcissism and self-reported mate poaching by including the Big Five personality variables and also examining poaching for the long-term (Studies 1 and 2) and for a new, exclusive relationship (Study 2).

Studies 3 and 4 experimentally investigated if grandiose narcissists reported more interest in targets with no relationship ties or targets who are already in a relationship. People who are already in relationships may be deemed more desirable, attracting grandiose narcissists. It is also possible that grandiose narcissists simply ignore others’ relationship statuses and pursue the person they find to be desirable and potentially available to them.

Given that the average effect size in personality and social psychology is *r* = .20, [31], we aimed to have at least 200 participants in each study to obtain adequate power (≥ .80). Moreover, material and data for all studies can be found in supplemental materials hosted on the Open Science Framework (OSF): https://osf.io/j2r8t/

## Study 1

We examined the extent to which grandiose narcissism was associated with mate poaching while controlling for Big Five variables. We used the Anonymous Romantic Attraction Survey from [19] seminal research on mate poaching. This survey assesses mate poaching for short-term sexual flings as well as long-term sexual affairs. Recent research focused on short-term mate poaching has demonstrated a link between grandiose narcissism and short-term mate poaching [18]. We sought to replicate and extend these findings by examining the association between grandiose narcissism and mate poaching for long-term sexual affairs as well; it is possible grandiose narcissists report long-term affairs as a means of demonstrating their power to take a mate away from someone else.

### Participants

Two-hundred forty-seven students (111 men, 133 women, and three who did not identify their sex) were recruited from Introductory Psychology courses in exchange for partial course credit (*M*_AGE_ = 19.48, *SD* = 3.66). 87% of the sample self-identified as Caucasian. 95.9% of these participants self-identified as heterosexual, 1.6% as gay, 2.4% as bisexual, and 0.8% did not indicate their sexual orientation.

### Methods and procedure

Participants reported to a research laboratory and first completed written informed consent. They then completed a series of questionnaires on a computer that assess personality and experiences with mate poaching.

#### Grandiose narcissism

Grandiose narcissism was assessed using the Narcissistic Personality Inventory (NPI) [4], which is a 40-item, forced-choice measure. Items on the NPI contain pairs of statements (e.g., “I am no better or no worse than most people;” “I think I am a special person”). A score of 0 is assigned to the non-narcissistic response and a score of 1 is assigned to the narcissistic response. Scores across the 40 items are summed so that higher scores represent higher levels of trait narcissism (For the present sample, α = .84, *M* = 17.13, *SD* = 6.92).

#### Big five personality

The Big Five Inventory (BFI) [32] consists of 44 items and is commonly used to measure neuroticism (e.g., “I see myself as someone who worries a lot;” α = .78; *M* = 2.97, *SD* = 0.78), extraversion (e.g., “I see myself as someone who is talkative;” α = .83; *M* = 3.49, *SD* = 0.74), openness to experience (e.g., “I see myself as someone who is curious about many different things;” α = .72; *M* = 3.57, *SD* = 0.58), conscientiousness (e.g., “I see myself as someone who does a thorough job;” α = .77; *M* = 3.61, *SD* = 0.60), and agreeableness (e.g., “I see myself as someone who is considerate and kind to almost everyone;” α = .72; *M* = 3.77, *SD* = 0.55) using 5-point scales, such that 1 = *disagree strongly* and 5 = *agree strongly*. Scores are computed by summing the items on each subscale; higher scores represent higher levels of each personality trait.

#### Mate poaching inventory

Mate poaching was assessed with the Schmitt and colleagues’ [19, 20] Anonymous Romantic Attraction Survey (ARAS). As in Schmitt and colleagues’ studies, one version of the questionnaire asked about short-term mate attraction experiences (e.g., hook-ups, one-night stands) and another asked about long-term mating experiences. Participants completed both versions, which were counterbalanced. The first question asked about the frequency of attempted mate poaching: “How often have you tried to attract someone who was already in a romantic relationship with someone else for a short-term sexual relationship (long-term sexual relationship) with you?” This question was assessed with a 7-point scale such that 1 = never and 7 = always (for short-term relationships, *M* = 2.06, *SD* = 1.28; for long-term relationships, *M* = 1.74, *SD* = 1.12). The second question was: “If you tried to attract someone who was already in a relationship for a short-term sexual relationship (long-term sexual relationship) with you, how successful have you been?” This question was answered using a 7-point scale such that 1 = not at all successful and 7 = very successful (for short-term relationships, *M* = 4.03, *SD* = 2.10, *n* = 143; for long-term relationships, *M* = 3.14, *SD* = 2.13, *n* = 138). The remaining items focus on being stolen away from an existing relationship and will not be discussed in this paper.

## Results

### Preliminary analyses

For short-term mate poaching, 69 men (62.16% of men) and 50 women (37.6% of women) indicated at least some experience with mate poaching (i.e., scored > 1). For long-term mate poaching, 46 men (41.4% of men) and 48 women (36% of women) indicated at least some experience with mate poaching (i.e., scored > 1). Men reported more frequent mate poaching attempts (*M* = 2.53, *SD* = 1.38) than women (*M* = 1.69, *SD* = 1.06) for the short-term, *t* (241) = 5.37, *p* < .001, Cohen’s *d* = 0.68. Men also reported more frequent mate poaching attempts (*M* = 1.91, *SD* = 1.26) than women (*M* = 1.61, *SD* = 0.98) for the long-term, *t* (242) = 2.10, *p* = .037, Cohen’s *d* = 0.26.

With respect to mate poaching success, women reported having the same success as men for short-term poaching attempts, *M*_women_ = 4.05, *SD* = 2.46; *M*_men_ = 4.05, *SD* = 1.75; *t* (114) = 0.01, *p* = .99, Cohen’s *d* = 0 and for long-term poaching attempts, *M*_women_ = 3.32, *SD* = 2.32; *M*_men_ = 2.97, *SD* = 1.92; *t* (136) = -0.96, *p* = .339, Cohen’s *d* = 0.16.

### Primary analyses

We first calculated the correlations among personality variables, mate poaching attempts, and mate poaching success for each sex. These results are summarized in Table 1. Grandiose narcissism was associated with more frequent short-term and long-term mate poaching attempts for men and for long-term mate poaching attempts for women. Women with higher grandiose narcissism also reported more success with poaching for a long-term relationship. Women with lower conscientiousness and lower openness to experience were more likely to report more frequent short-term mate poaching.

**Table 1:** Correlations between mate poaching measures and personality variables in men and women (Study 1).

Personality Measures:	Men vs Women	Short-Term Poaching Attempts	Short-Term Poaching Success	Long-Term Poaching Attempts	Long-Term Poaching Success
Grandiose Narcissism	M	.265**	.003	.237*	.138
	W	.077	-.030	.255**	.289*
Extraversion	M	.150	-.216^#^	.103	.016
	W	.093	-.082	.143	-.006
Agreeableness	M	-.087	-.044	-.127	-.104
	W	-.083	-.081	.006	.025
Conscientiousness	M	-.049	-.057	-.071	-.110
	W	-.196*	-.164	-.133	-.112
Neuroticism	M	-.099	-.108	.035	-.028
	W	-.039	.204^#^	-.141	.173
Openness	M	-.058	-.039	.070	-.029
	W	-.250**	-.240^#^	-.033	.091

^#^*p* < .10

*p < .05

**p < .01

We next regressed mate poaching variables on grandiose narcissism, while controlling for the Big Five personality variables for each sex. Given that the sample sizes for mate poaching success variables were small, we use caution in our interpretation of the results for these variables. See Table 2 for a summary of results.

**Table 2:** Regression analyses of personality variables predicting mate poaching in men and women (Study 1).

Predictors:		Short-Term Poaching Attempts		Short-Term Poaching Success		Long-Term Poaching Attempts		Long-Term Poaching Success		
		M (*n* = 111)	W (*n* = 132)	M (*n* = 70)	W (*n* = 46)	M (*n* = 111)	W (*n* = 133)	M (*n* = 47)		W (*n* = 45)
Grandiose Narcissism	β	.259*	.208^#^	.093	.249	.218^#^	.306**	.120		.340*
	*pr*	.222	.174	.080	.220	.183	.242	.102		.307
Extraversion	β	.126	.045	-.276*	-.088	.112	-.010	-.018		-.028
	*pr*	.123	.038	-.265	-.081	.107	-.008	-.018		-.026
Agreeableness	β	-.050	-.079	-.014	.110	-.105	.043	-.128		.132
	*pr*	-.045	-.074	-.013	.103	-.092	.039	-.103		.129
Conscientiousness	β	-.126	-.140	-.165	-.072	-.107	-.141	-.053		-.114
	*pr*	-.124	-.144	-.159	-.074	-.102	-.139	-.050		-.120
Neuroticism	β	-.153	.007	-.188	.311*	-.017	-.040	-.090		.325*
	*pr*	-.136	.007	-.170	.284	-.015	-.037	-.077		.297
Openness	β	-.152	-.384***	-.032	-.381**	.018	-.151	-.124		.002
	*pr*	-.153	-.367	-.032	-.361	.018	-.148	-.113		.002
*R*^2^		.145	.173	.117	.207	.095	.105	.050		.183
Cohen’s *f*^2^		.169	.209	.132	.261	.105	.117	.053		.224

^#^*p* < .10

*p < .05

**p < .01

***p < .001

Grandiose narcissism predicted more frequent short-term mate poaching attempts while controlling for the Big Five personality factors for both men (*β* = .259, *t* = 2.108, *p* = .038) and women (*β* = .208, *t* = 1.851, *p* = .067). For women, openness to experience was also significant; women who were less open to experience reported more frequent short-term mate poaching attempts. For short-term mate poaching success, grandiose narcissism was not a significant predictor for men (*β* = .093, *t* = 0.614, *p* = .541) or women (*β* = .249, *t* = 1.576, *p* = .121). However, for men, higher extraversion was associated with less success at poaching for the short-term, and for women, there significant effects for openness to experience and neuroticism indicating that more neurotic and less open women reported more frequent short-term mate poaching success.

For long-term mate poaching, grandiose narcissism was a significant predictor for women (*β* = .306, *t* = 2.623, *p* = .010) and a marginally significant predictor for men (*β* = .218, *t* = 1.725, *p* = .088). None of the Big Five variables were significant. Grandiose narcissism was not a significant predictor of success at poaching for the long-term for men (*β* = .120, *t* = 0.718, *p* = .476), but it was for women (*β* = .340, *t* = 2.369, *p* = .021). None of the other variables were significant except for women’s neuroticism; when women were more neurotic, they reported more success at poaching for a long-term sexual relationship.

## Discussion

Consistent with past research, grandiose narcissism appears to be an important variable implicated in short-term mate poaching attempts—even while controlling for other relevant variables [18]. It seems that grandiose narcissists report seeking to increase their mating success by attempting to gain access to mates who are not available for short-term flings. Also, consistent with Kardum and colleagues, grandiose narcissism was not associated with success at short-term mate poaching. Although grandiose narcissists tend to be attractive [33] and are perceived as sexy [22], perhaps their exploitative, self-serving, and promiscuous behavior does not provide enough potential benefits to the poached to incur the costs of infidelity.

Expanding on past research, Study 1 also revealed that grandiose narcissism was associated with mate poaching attempts for long-term sexual relationships as well. This effect was stronger for women, who also reported success at mate poaching. Thus, it does not appear that grandiose narcissistic women are only looking for a short-term fling when they make themselves sexually available to others. Rather, it appears that they are also inclined to form longer-term entanglements as well.

The purpose of Study 2 was to replicate the results of Study 1 with an improved measure of mate poaching. There are two main criticisms of the Anonymous Romantic Attraction Survey [27]. First, it does not make it clear that the person knew that the target of mate poaching was already in a relationship. Second, the meaning of the response scale is vague. For example, if somebody indicates they “frequently” engage in mate poaching, does this mean that they engaged in mate poaching two or three times or two or three hundred times? To account for these issues in Study 2, we used a measure by Davies and colleagues [27] that clarifies that in order for someone to mate poach, he or she must be aware that the target is already in a relationship. Additionally, the response scale is improved by asking participants to be more specific about the number of times they have been involved in mate poaching. Moreover, in addition to assessing poaching for a short-term and long-term sexual relationship, an item on this questionnaire also inquires about mate poaching to form a new permanent relationship. Previous research found a correlation between grandiose narcissism and mate poaching using this questionnaire [17], but their study did not control for Big Five personality.

## Study 2

### Participants

Two-hundred thirty-five students (105 men and 130 women) were recruited from Introductory Psychology courses in exchange for partial course credit (*M*_AGE_ = 20.23 years, *SD* = 6.41). 82.3% of participant self-identified as Caucasian. 85.2% of participants self-identified as heterosexual, 11.0% identified as gay, 1.7% identified as bisexual, and 2.1% either identified as “other” or did not specify their sexual orientation.

### Materials and procedure

As in Study 1, participants first completed written informed consent and were then asked to complete the Narcissistic Personality Inventory (NPI [4] α = .86, *M* = 15.73, *SD* = 7.21) and the Big Five Inventory (BFI) [32]. For neuroticism, α = .78; *M* = 2.96, *SD* = 0.75; for extraversion, α = .84, *M* = 3.41, *SD* = 0.75; for openness to experience, α = .74, *M* = 3.55, *SD* = 0.59; for conscientiousness, α = .74; *M* = 3.51, *SD* = 0.56; and for agreeableness, α = .72, *M* = 3.76, *SD* = 0.53.

#### Mate poaching questionnaire

To assess the extent to which participants have engaged in mate poaching behavior, we used items from the Davies, Shackelford, and Hass [27] questionnaire. The introduction of this questionnaires states, “A mate poacher is someone who has sexual relations with a person whom the mate poacher knows is already in a nominally exclusive relationship with someone else. An exclusive relationship is one in which a couple has an understanding that their relationship is sexually monogamous, and so sexual relations with people outside the relationship is a violation of the relationship. Exclusive relationships therefore do not include so-called ‘open’ relationships.” Following these statements was the assessment of mate poaching. To assess short-term mate poaching, the item was, “Knowing from the start that a person was already in an exclusive relationship, have you ever attempted to attract this person as a short-term sexual partner?” Similar items were worded for a long-term sexual affair (more than one sexual encounter) and a new exclusive relationship (permanently abandoning their relationship and to start a new exclusive relationship). Responses were made such that 0 = *never*, 1 = once, 2 = twice, 3 = three times, 4 = four or more times. For each type of mate poaching, participants were asked how successful they had been (0 = not at all successful, 4 = very successful).

## Results

### Preliminary analyses

For short-term mate poaching, 48 men (45.7% of men) and 50 women (38.5% of women) indicated at least some experience with mate poaching (i.e., scored > 0). For long-term mate poaching, 24 men (22.8% of men) and 44 women (33.8% of women) indicated at least some experience with mate poaching (i.e., scored > 0). When asked about poaching for an exclusive relationship, 38 men (36.2%) and 44 women (33.8%) indicated at least some experience with mate poaching (i.e., scored > 0).

Men and women did not differ in their reports of mate poaching attempts or their success at mate poaching (short-term poaching attempt: *t* (233) = 0.91, *p* = .366; long-term poaching attempt: *t* (233) = -1.385, *p =* .167; exclusive relationship poaching attempt: *t* (233) = .022, *p =* .983; short-term poaching success: *t* (96) = -1.50, *p* = .137, long-term poaching success *t* (66) = 0.50, *p* = .618; new relationship poaching success: *t* (80) *= -*0.545, *p* = .587.

### Primary analyses

We first calculated the correlations among personality variables, mate poaching attempts, and mate poaching success for each sex. Again, we note that the sample sizes for mate poaching success variables were small and use caution in our interpretation of these results. Results are summarized in Table 3. Grandiose narcissism was not correlated with any mate poaching variables for men but was associated with mate poaching attempts for women. Grandiose narcissism was also associated with women’s reported success at poaching for a long-term affair. Women’s extraversion, lower agreeableness, and lower conscientiousness were associated with their reported short-term mate poaching attempts. Women’s lower agreeableness was also associated with their reported attempts at poaching for a long-term affair and for an exclusive relationship.

**Table 3:** Correlations between mate poaching measures and personality variables in men and women (Study 2).

Personality measures:	Men vs Women	Short-Term Poaching Attempts	Short-Term Poaching Success	Long-Term Poaching Attempts	Long-Term Poaching Success	Exclusive Relationship Poaching Attempts	Exclusive Relationship Poaching Success
Grandiose Narcissism	M	.151	-.015	.086	-.014	.065	.025
	W	.289**	.143	.288**	.322*	.212*	.121
Extraversion	M	.125	.078	.124	.188	.150	.005
	W	.201*	.244^#^	.114	.306*	.107	.061
Agreeableness	M	-.142	.007	-.185^#^	-.191	-.173^#^	-.269
	W	-.219*	-.137	-.266*	-.115	-.240**	.201
Conscientiousness	M	-.010	-.022	-.117	-.097	.037	-.213
	W	-.261**	.000	-.142	.175	-.075	.385**
Neuroticism	M	-.041	-.059	-.061	.135	-.182^#^	-.144
	W	.118	.164	.067	-.199	.051	-.259^#^
Openness	M	.030	-.059	.089	.051	.155	.219
	W	-.084	-.206	.012	.032	.072	.110

^#^*p* < .10

*p < .05

**p < .01

To examine whether grandiose narcissism predicted mate poaching, we regressed mate poaching variables on grandiose narcissism, controlling for the Big Five personality variables for each sex. See Tables 4 and 5 for a summary of results. Grandiose narcissism was associated with more frequent short-term (*β* = .293, *t* = 33.069, *p* = .003) and long-term mate poaching attempts (*β* = .267, *t* = 2.708, *p* = .008) for women, but not men (short-term: *β* = .069, *t* = 0.516, *p* = .607; long-term: (*β* = -.051, *t* = -0.385, *p* = .701). A marginally significant result emerged for men attempting to poach a partner for an exclusive relationship, such that grandiose narcissism was related to fewer mate poaching attempts (*β* = -.208, *t* = -1.658, *p* = .100). Grandiose narcissism was not associated with mate poaching attempts for an exclusive relationship among women (*β* = .149, *t* = 1.476, *p* = .142) Women with lower conscientiousness were more likely to report more frequent short-term mate-poaching attempts, and women with lower agreeableness reported less frequent long-term mate poaching attempts. Both men and women with lower agreeableness reported more frequent mate poaching attempts for an exclusive new relationship.

**Table 4:** Regression analyses of personality variables predicting mate poaching in men and women (Study 2).

Predictors:		Short-Term Poaching Attempts		Short-Term Poaching Success		Long-Term Poaching Attempts		Long-Term Poaching Success	
		M (*n* = 105)	W (*n* = 130)	M (*n* = 48)	W (*n* = 50)	M (*n* = 105)	W (*n* = 130)	M (*n* = 24)	W (*n* = 44)
Grandiose Narcissism	β	.069	.293**	-.130	.053	-.051	.267**	-.184	.133
	*pr*	.052	.267	-.092	.046	-.039	.237	-.144	.122
Extraversion	β	.069	.117	.159	.286^#^	.130	.003	.308	.220
	*pr*	.054	.115	.117	.262	.104	.003	.251	.204
Agreeableness	β	-.166	-.029	-.040	-.068	-.201^#^	-.209*	-.206	-.274
	*pr*	-.138	-.025	-.034	-.056	-.169	-.174	-.182	-.245
Conscientiousness	β	.040	-.249**	-.006	.028	-.061	-.105	.069	.139
	*pr*	.035	-.248	-.005	.025	-.054	-.104	.058	.135
Neuroticism	β	-.039	.114	-.062	.229	-.115	-.015	.150	-.185
	*pr*	-.036	.103	-.055	.192	-.106	-.013	.130	-.163
Openness	β	.021	-.081	-.065	.010	.092	.018	.002	-.030
	*pr*	.022	-.086	-.064	.009	.094	.018	.002	-.031
*R*^2^		.044	.200	.022	.124	.071	.147	.114	.202
Cohen’s *f*^2^		.046	.250	.022	.141	.076	.172	.129	.253

^#^*p* < .10

*p < .05

**p < .01

**Table 5:** Regression analyses of personality variables predicting mate poaching in men and women (Study 2).

Predictors:		Exclusive Relationship Poaching attempts		Exclusive Relationship Success	
		M (*n* = 105)	W (*n* = 130)	M (*n* = 38)	W (*n* = 44)
Grandiose Narcissism	β	-.208^#^	.149	-.277	.135
	*pr*	-.166	.132	-.209	.125
Extraversion	β	.202^#^	.033	.004	-.018
	*pr*	.167	.031	.004	-.017
Agreeableness	β	-.351**	-.247*	-.361	.045
	*pr*	-.301	-.199	-.266	.043
Conscientiousness	β	.143	-.031	-.089	.328^#^
	*pr*	.132	-.030	-.070	.307
Neuroticism	β	-.254*	-.038	-.472*	-.135
	*pr*	-.241	-.032	-.389	-.135
Openness	β	.177^#^	.085	.302^#^	.016
	*pr*	.188	.085	.309	.016
*R*^2^		.160	.101	.254	.187
Cohen’s *f*^2^		.190	.112	.340	.230

^#^*p* < .10

*p < .05

**p < .01

Grandiose narcissism was not associated with mate poaching success among men (short-term: *β* = -.130, *t* = -0.594, *p* = .556; long-term: *β* = -.184, *t* = -0.602, *p* = .555; exclusive: *β* = -.277, *t* = -1.172, *p* = .250) or women (short-term: *β* = .053, *t* = 0.301, *p* = .765; long-term: *β* = .133, *t* = 0.745, *p* = .461; exclusive: *β* = .135, *t* = 0.767, *p* = .448). With respect to mate poaching success, men with lower neuroticism were more likely to report success at poaching for an exclusive new relationship. None of the other variables were statistically significant.

## Discussion

The results from Study 2 revealed that grandiose narcissistic women reported more frequent attempts at mate poaching; this does not appear to be the case for the formation of new exclusive relationships. Thus, grandiose narcissistic women’s mate poaching attempts appear to have more to do with casual sex than with a desire to form lasting emotional ties, even though there are greater social sanctions for their promiscuous sexual behavior than there are for men [34].

Of interest, grandiose narcissism was not associated with mate poaching among men. This result is consistent with results reported by Kardum and colleagues [18]. Thus, it is possible that grandiose narcissistic women are more frequently guilty of mate poaching.

One issue that is worth noting is that although we obtained data suggesting that one-third to almost two-thirds of our participants reported having experience with mate poaching in Studies 1 and 2, not everybody attempts to mate poach. Thus, it is possible that random responding could make correlations appear stronger than they are [35].

Although there is considerable evidence that grandiose narcissists report greater lifetime prevalence of mate poaching attempts, their actual behavior remains unknown in specific situations where mate poaching is a possibility. Do grandiose narcissists experiencea greater attraction to potential mates who are already in a relationship? There is a growing amount of evidence to suggest that people tend to be more interested in relationships with potential mates when these potential mates are already paired, probably because these existing ties are indicative of higher mate quality [1]. Thus, if partnered mates are deemed as more desirable mates, then there should be an indication of a preference for potential partners who are known to be in relationships. In particular, we would expect a pattern of results showing that a) grandiose narcissists are more interested in potential mates who are already partnered, and b) their preference would be for shorter term sexual relationships rather than longer-term relationships.

We examine this question in Study 3 by using an attraction paradigm that we modified from a study conducted by Parker and Burkley [36]. Parker and Burkley asked participants to complete a series of questionnaires like the ones a person would expect to find on dating websites such as eHarmony.com or match.com. After completing these questionnaires, participants were led to believe that the computer was matching them to another student on campus who gave similar responses. Participants were randomly assigned to read that the target was single or in a current relationship. Participants were then asked how likely they would be to show interest in the target (by making eye contact and smiling), how compatible they think the person was, how likely they would be to initiate a conversation, how likely they would be to initiate a relationship, and how direct they would be in initiating a romantic relationship. Parker and Burkley combined these items into a measure of pursuit of the target. They also assessed the extent to which they found the target to be physically attractive. This is important because one can find a person to be attractive and yet not express interest in the person for a relationship.

Parker and Burkley [36] reported that men found the target to be more physically attractive than women found the target. For participants who were in a relationship themselves, attached men were more interested in the target than attached women were, but there was no effect for the relationship status of the target. For single participants, a different pattern emerged. Single men were more interested in the target overall than single women, and showed no difference in interest between an attached and single target. Single women, on the other hand, were more interested in pursuing an attached target than a single target. Parker and Burkley concluded that women were more likely to mate poach than men.

In the present study, we made some modifications to Parker and Burkley’s [36] paradigm by directly asking participants if they would be interested in the target for a relationship and if they would “make out” with the participant. We included measures of grandiose narcissism and Big Five personality to their paradigm to examine the extent to which grandiose narcissism was involved in pursuing a target who was already in a relationship (while controlling for Big Five variables).

## Study 3

### Participants

Two hundred and forty-nine (118 men and 131 women) participants were recruited from Introductory Psychology courses in exchange for partial course credit (*M*_AGE_ = 19.69, *SD* = 3.81). Four participants were excluded because they indicated that they were gay (one man and three women) and the computer program used in the study would not allow us to account for lesbian or gay male sexual orientation. The majority (70.7%) of the participants self-identified as Caucasian. 107 participants (43%) indicated they were already in a romantic relationship.

### Materials and procedure

Upon arriving to the laboratory, participants completed written informed consent. Participants were seated at a computer and asked to complete a questionnaire, which included an assessment of personality and questions assessing romantic partner compatibility. As in the previous studies, grandiose narcissism was assessed with the Narcissistic Personality Inventory (NPI [4] α = .86, *M* = 15.97, *SD* = 7.20) and Big Five personality traits with the Big Five Inventory (BFI) [32]. For neuroticism, α = .82; *M* = 2.77, *SD* = 0.80; for extraversion, α = .84, *M* = 3.46, *SD* = 0.72; for openness to experience, α = .75, *M* = 3.55, *SD* = 0.58; for conscientiousness, α = .76; *M* = 3.67, *SD* = 0.56; and for agreeableness, α = .73, *M* = 3.97, *SD* = 0.51.

The questions to assess relationship partner compatibility were similar to what one might find on eHarmony.com or match.com. This questionnaire was not used for analysis as it was part of the cover story. Participants were told that the information they provided would be used to match them up with someone on campus who has similar interests. Finally, sex, age, and relationship status were assessed.

Following the procedure outlined by Parker and Burkley [36], participants were next shown a picture of a target individual and told that based on their previous answers, they have “similar interests” with this target. The women viewed a picture of the male target and the men viewed a picture of a female target. These pictures were pre-tested by a separate sample for their level of attractiveness using a 10-point scale, such that 1 = very unattractive and 10 = very attractive. The mean rating for the male photo was 6.62 (*SD* = 1.86) and the mean rating for female photo was 7.03 (*SD* = 1.29). Participants were randomly assigned to a description of the target as “single” or “in a relationship” and were then asked to take a short survey on the level of interest they have in the target. Like Parker and Burkley, we also asked participants a series of questions about the participant’s interest in the target, but our dependent variables were only the items that assessed mate poaching specifically. Responses were made on 5-point scales such that 1 = very unlikely and 5 = very likely. The items were a) “How likely would you be to pursue this individual for a relationship?” (*M* = 2.43, *SD* = 1.28), and b) “How likely would you be to make out with this individual?” (*M* = 2.38, *SD* = 1.34). We also assessed the extent to which the participant found the target attractive using a 5-point scale such that 1 = very unattractive and 5 = very attractive (*M* = 3.76, *SD* = 1.15).

## Results

We first centered all variables prior to computing analyses. We then computed correlations between predictor/control variables (the Big Five variables) and outcome variables, which are displayed in Table 6. Grandiose narcissism was correlated with a greater likelihood of making out with the target, but not with finding the target attractive or pursuing the target for a relationship.

**Table 6:** Correlations between predictor variables and mate poaching outcome variables (Study 3).

	Attractive	Pursue for Relationship	Make Out
Grandiose Narcissism	.078	.089	.249***
Extraversion	.052	.098	.168*
Agreeableness	-.004	.073	.031
Conscientiousness	-.034	-.017	-.016
Neuroticism	-.137*	-.082	-.184**
Openness	.026	.078	.078
Participant Sex (-.5 = female, .5 = male)	.485***	.235***	.374***
Participant Relationship Status (-.5 = Single, .5 = Attached)	-.180**	-.450	-.395***
Target Relationship Status (-.5 = Single, .5 = Attached)	-.003	-.075	-.053

*p < .05

**p < .01

***p < .001

Participant Sex was coded -.5 = female, .5 = male; Participant Relationship Status was coded -.5 = single, .5 = attached; Target Relationship Status was coded -.5 = single and .5 = attached.

We then entered variables into a regression model; primary variables of participants’ relationship status, target relationship status, sex, and NPI scores were entered into Step 1 (controlling for Big Five variables), all two-way interactions among primary variables were entered into Step 2, all three-way interactions among primary variables were entered into Step 3, and the four-way interaction among primary variables was entered into Step 4. Given that none of the four-way interactions reached statistical significance, Step 3 analyses are reported in Table 7.

**Table 7:** Regression analyses predicting mate poaching variables (Study 3).

	Attractive β (*pr*)	Pursue for Relationship β (*pr*)	Make Out β (*pr*)
Grandiose Narcissism	-.067 (-.061)	.006 (.005)	.107 (.103)
Extraversion	.105 (.098)	.124^#^ (.118)	.122^#^ (.121)
Agreeableness	-.024 (-.023)	.091 (.088)	.021 (.022)
Conscientiousness	.008 (.008)	-.003 (-.003)	-.029 (-.031)
Neuroticism	.056 (.047)	.080 (.069)	-.004 (-.004)
Openness	-.048 (-.054)	.021 (.024)	-.019 (-.023)
Participant Sex (-.5 = female, .5 = male)	.495***(.449)	.206**(.209)	.309***(.317)
Participant Relationship Status (-.5 = Single, .5 = Attached)	-.122*(-.134)	-.468***(-.469)	-.381***(-.411)
Target Relationship Status (-.5 = Single, .5 = Attached)	-.004 (-.004)	-.028 (-.032)	-.022 (-.026)
Grandiose Narcissism × Target Relationship Status	-.052 (-.056)	.068 (.075)	.034 (.040)
Grandiose Narcissism × Sex	.018 (.020)	.076 (.085)	.161**(.185)
Grandiose Narcissism × Participant Relationship Status	.021 (.022)	-.001 (-.001)	-.016 (-.018)
Target Relationship Status × Sex	.029 (.032)	-.053 (-.059)	.020 (.023)
Target Relationship Status × Participant Relationship Status	.057 (.064)	.018 (.021)	-.002 (-.002)
Sex × Participant Relationship Status	-.021 (-.023)	-.044 (-.049)	-.087 (-.102)
Grandiose Narcissism × Target Relationship Status × Participant Relationship Status	-.019 (-.020)	.044 (.048)	.050 (.057)
Grandiose Narcissism × Target Relationship Status × Sex	.029 (.031)	.029 (.032)	.042 (.048)
Grandiose Narcissism × Participant Relationship Status × Sex	-.049 (-.052)	.162**(.175)	.083 (.094)
Target Relationship Status × Participant Relationship Status × Sex	-.015 (-.017)	-.016 (-.019)	-.063 (-.074)
*R*^2^	.266	.277	.345
Cohen’s *f*^2^	.362	.383	.527

^#^*p* < .10

*p< .05

** *p*< .01

*** *p*< .001

Participant Sex was coded -.5 = female, .5 = male; Participant Relationship Status was coded -.5 = single, .5 = attached; Target Relationship Status was coded -.5 = single and .5 = attached. Significant results are emphasized in **bold**.

For finding the target attractive, only main effects for sex and a participants’ relationship status were significant. Men were more likely to find the target attractive than women, and single people were more likely to find the target attractive than attached people. Grandiose narcissism was not a predictor of finding the target attractive in general (*β* = -.067, *t* = -0.913, *p* = .362) and grandiose narcissists did not find attached targets as more attractive than unattached targets (*β* = -.052, *t* = -0.842, *p* = .401).

**Fig 1:**
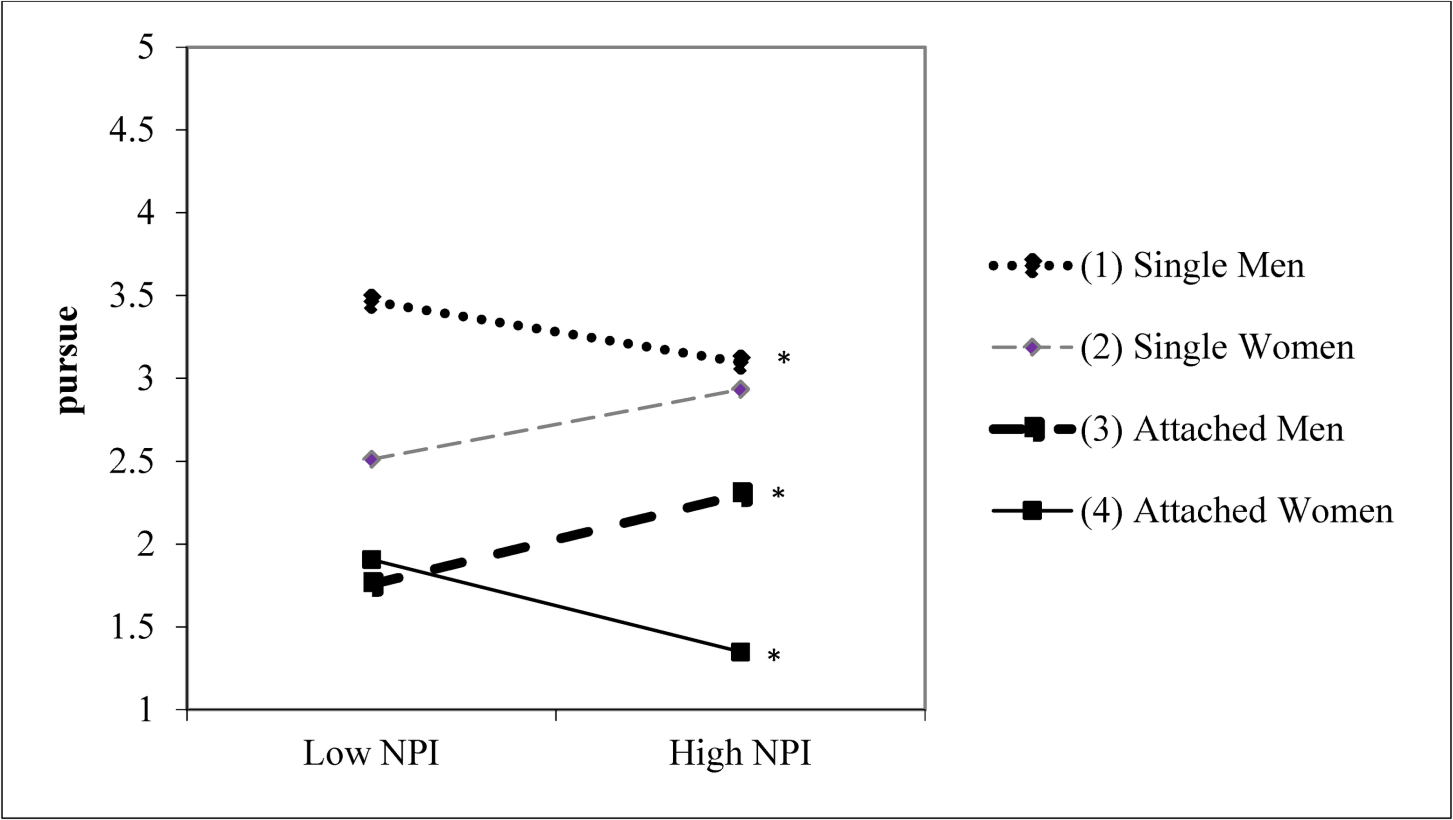
Narcissism × participant relationship status × sex for the likelihood of pursuing the target for a relationship. Asterisks indicate that the simple slopes for single men attached men, and attached women were statistically significant. (Study 3).

There was a significant three-way interaction between grandiose narcissism, participant relationship status, and participant sex for the likelihood of pursuing the target for a relationship (*β* = .162, *t* = 2.666, *p* = .008). This three-way interaction is displayed in Fig 1. Data are plotted at ±1 *SD* from the mean of grandiose narcissism. The simple slope for attached men was significant (simple slope = .05, *t* = 9.24, *p* < .001), revealing that men in a relationship were more likely to pursue the target for a relationship at higher levels of grandiose narcissism. The simple slope for attached women (simple slope = -.04, *t* = -3.27, *p* = .001) revealed that women in a relationship were less likely to pursue the target for a relationship at higher levels of grandiose narcissism. The simple slope for single men was also significant (simple slope = -.02, *t* = -2.38, *p* = .02), revealing that single men were less interested in pursuing the target for a relationship at higher levels of grandiose narcissism. The simple slope for single women was not significant (simple slope = .024, *t* = 1.56, *p* = .12). With the exception of the slope differences between single women and attached men (*t* = -1.65, *p* = .10), the differences in slopes were all significant (*t*s ≥ -2.16, *p*s ≤ .03). The grandiose narcissism × target relationship status interaction was not significant (*β* = .068, *t* = 1.127, *p* = .261), indicating that grandiose narcissism was not associated with mate poaching for a long-term affair.

**Fig 2:**
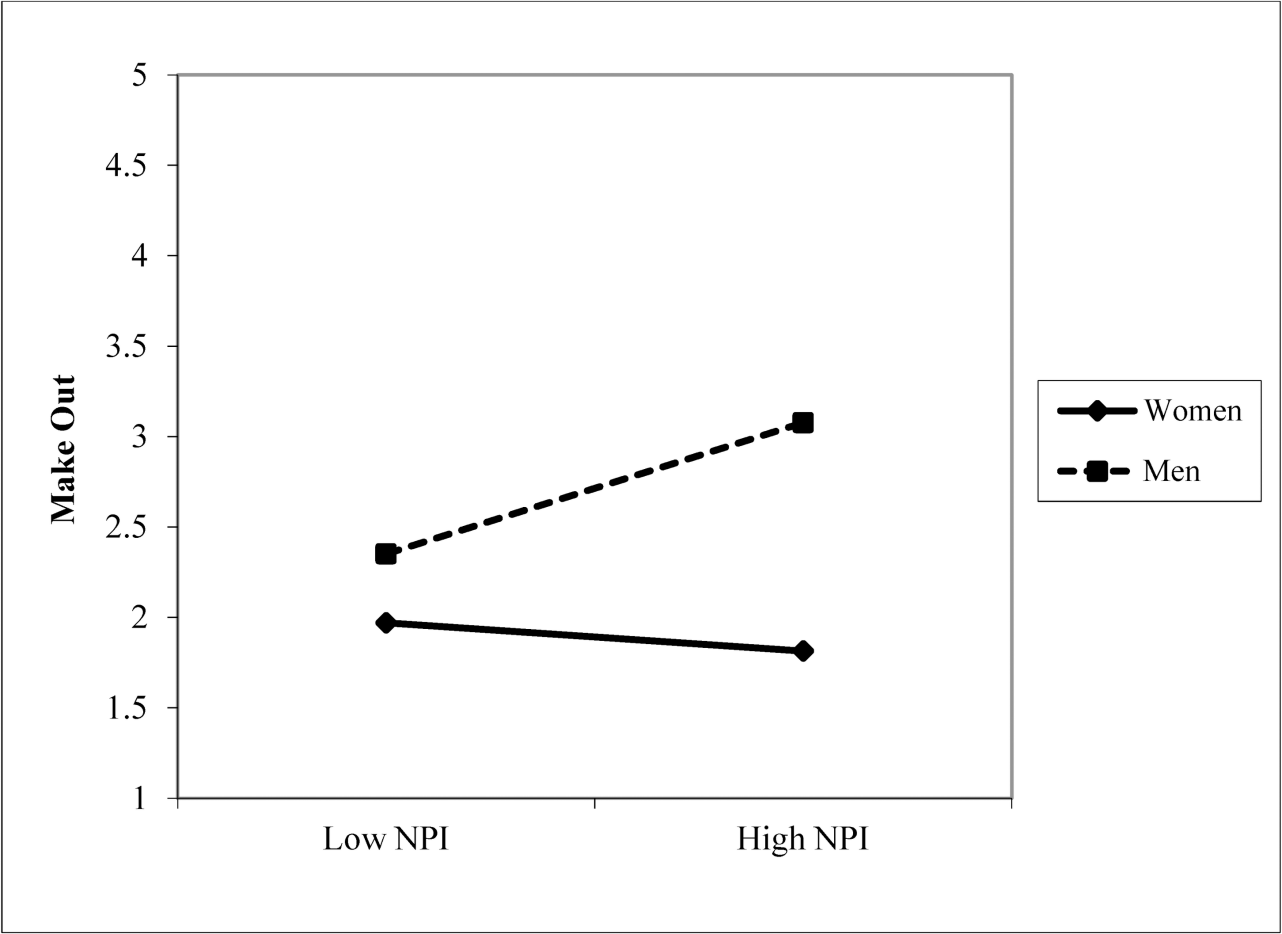
Narcissism × sex for the likelihood of making out with the target. (Study 3).

For the likelihood of making out with the target, there was a significant interaction between grandiose narcissism and participant sex, *β* = .161, *t* = 2.819, *p* = .005. This interaction is displayed in Fig 2. The simple slopes for men (simple slope = 0.051., *t* = .007, *p =* .995) and women (simple slope = -0.011., *t* = -0.002, *p =* .999) were not significant. Grandiose narcissists did not express a greater likelihood of making out with an attached target over a single one (*β* = .068, *t* = 1.127, *p* = .261), suggesting that grandiose narcissism was not associated with mate poaching for a short-term affair.

## Discussion

A theme of these analyses is that the relationship status of the target did not play a role in participants’ judgments about their likelihood of pursuing the target for short-term or long-term sexual relationships. Thus, it is likely people are simply interested in the target and not necessarily concerned that the target is in a relationship. Results of Study 3 do suggest that grandiose narcissism plays a role in pursuing a target, especially for men looking for a short-term relationship. However, grandiose narcissists do not report a greater likelihood of pursuing a target in a relationship; attached men were more likely to indicate they would cheat on their romantic partner when they were grandiose narcissistic but not necessarily to pursue someone in a relationship.

We developed Study 4 to improve upon Study 3. We added more realism to the study paradigm by leading participants to believe that we were piloting a campus-wide dating service. As in Study 3, participants completed the personality measures and dating profile-type questions. They were then randomly assigned to a profile of a target whose relationship status was listed as single or in a relationship. As in Study 3, we asked participants how attractive they found the target. We also asked participants about the extent to which they would pursue the target for a relationship. We revised the question for assessing short-term mating to ask how likely participants would be to “hook up” with the target to make the language consistent with the language of college students. Additionally, we added a behavioral measure at the end of the study assessing the participant’s intention to pursue the target by entering a raffle for a free date [37].

## Study 4

### Participants

Two hundred forty (108 men and 132 women) participants were recruited from Introductory Psychology courses in exchange for partial course credit (*M*_AGE_ = 19.30, *SD* = 3.73). Seven of these participants were excluded because they indicated that they were gay (three men and four women) and the computer program used in the study would not allow us to account for lesbian or gay male sexual orientation. The majority (77.7%) of the participants self-identified as Caucasian. Ninety-nine participants (42.5%) indicated they were already in a romantic relationship.

### Materials and procedure

As in Study 3, participants completed written informed consent upon arriving to the laboratory and were then seated at a computer. They were asked to complete a questionnaire, which included an assessment of personality and questions assessing romantic partner compatibility. As in the previous studies, grandiose narcissism was assessed with the Narcissistic Personality Inventory (NPI [4] ; α = .84, *M* = 15.26, *SD* = 6.56) and Big Five personality traits with the Big Five Inventory (BFI) [32]. For neuroticism, α = .80; *M* = 2.94, *SD* = 0.79; for extraversion, α = .87, *M* = 3.34, *SD* = 0.77; for openness to experience, α = .78, *M* = 3.60, *SD* = 0.58; for conscientiousness, α = .74; *M* = 3.68, *SD* = 0.56; and for agreeableness, α = .71, *M* = 3.91, *SD* = 0.51.

The questions to assess relationship partner compatibility were the same as Study 3. Also, as in Study 3, participants were informed that their information would be used to match them up with someone on campus who had similar interests. Likewise, we assessed sex, age, and relationship status.

Participants were next shown a dating profile of a target individual and told that they had been “paired with a student who shares similar attributes and interests.” The women viewed the picture of the male target and the men viewed the picture of the female target that were used in Study 3. As part of the profile, the participants were randomly assigned to a description of the target as “single” or “in a current relationship.” All remaining aspects of the profile were identical. The participants were asked to take the short survey about their level of interest in the target. Responses were made on 5-point scales such that 1 = very unlikely and 5 = very likely. The items were a) “How likely would you be to pursue this individual for a relationship?” (*M* = 2.31, *SD* = 1.18), and b) “How likely would you be to hook up with this individual?” (*M* = 2.30, *SD* = 1.34). We also assessed the extent to which the participant found the target “physically appealing” using a 5-point scale such that 1 = not at all appealing and 5 = very appealing (*M* = 3.66, *SD* = 0.93).

At the end of the survey, participants were offered the opportunity to enter a raffle to win a free date. If they were interested, they were told to fill out a raffle ticket. A research assistant surreptitiously recorded whether or not a participant completed the raffle and then immediately destroyed any identifying information.

## Results

Prior to computing analyses, we first centered all variables. First, we computed correlations between predictor/control variables (the Big Five variables) and outcome variables, which are displayed in Table 8. Grandiose narcissism was correlated with a greater likelihood of hooking up with the target. There was a marginally significant correlation between grandiose narcissism and finding the target attractive. Grandiose narcissism was not associated with pursuing the target for a relationship.

**Table 8:** Correlations between predictor variables and mate poaching outcome variables (Study 4).

	Physically Appealing	Pursue for Relationship	Hook Up
Grandiose Narcissism	.115	.081	.235***
Extraversion	.000	-.010	.034
Agreeableness	.018	.034	-.055
Conscientiousness	-.022	-.093	-.138*
Neuroticism	-.089	-.108	-.084
Openness	.006	-.057	-.032
Participant Sex (-.5 = female, .5 = male)	.420***	.438***	.587***
Participant Relationship Status (-.5 = Single, .5 = Attached)	.121^#^	-.323***	.314***
Target Relationship Status (-.5 = Single, .5 = Attached)	-.026	-.006	-.019

^#^*p* < .10

*p < .05

*** *p* < .001

Participant Sex was coded -.5 = female, .5 = male; Participant Relationship Status was coded -.5 = single, .5 = attached; Target Relationship Status was coded -.5 = single and .5 = attached.

We then entered variables into a regression model as we did in Study 3. Given that none of the four-way interactions reached statistical significance, Step 3 analyses are reported in Table 9. For finding the target physically appealing, a significant main effect for sex was found; men were more likely to find the target physically appealing than women. Grandiose narcissism was not associated with finding the target to be physically appealing (*β* = .107, *t* = 1.418, *p* = .158). Likewise, grandiose narcissists did not find the target in a relationship to be more or less physically appealing than the single target (*β* = .091, *t* = 1.424, *p* = .156).

**Table 9:** Regression analyses predicting mate poaching variables (Study 4).

	Physically Appealing β (*pr*)	Pursue for Relationship β (*pr*)	Hook Up β (*pr*)
Grandiose Narcissism	.107 (.097)	.123^#^ (.120)	.264***(.287)
Extraversion	.003 (.003)	.039 (.039)	.040 (.046)
Agreeableness	.075 (.068)	.096 (.094)	.039 (.045)
Conscientiousness	.015 (.014)	-.055 (-.056)	-.083 (.099)
Neuroticism	.063 (.054)	.014 (.013)	.121^#^ (.130)
Openness	-.047 (-.049)	-.148*(-.163)	-.118*(-.152)
Participant Sex (-.5 = female, .5 = male)	.444***(.391)	.390***(.373)	.559***(.558)
Participant Relationship Status (-.5 = Single, .5 = Attached)	.014 (.015)	-.244***(-.259)	-.166**(-.209)
Target Relationship Status (-.5 = Single, .5 = Attached)	-.042 (.042)	.001 (.001)	-.008 (-.010)
Grandiose Narcissism × Target Relationship Status	.091 (.097)	.107^#^ (.123)	.125*(.167)
Grandiose Narcissism × Sex	.047 (.049)	.057 (.063)	.137**(.175)
Grandiose Narcissism × Participant Relationship Status	.054 (.056)	.083 (.093)	-.017 (-.022)
Target Relationship Status × Sex	-.071 (-.072)	-.131*(-.142)	-.125*(-.157)
Target Relationship Status × Participant Relationship Status	-.106 (-.107)	.013 (.014)	.033 (.042)
Sex × Participant Relationship Status	-.003 (-.003)	.001 (.001)	-.003 (-.004)
Grandiose Narcissism × Target Relationship Status × Participant Relationship Status	.049 (.051)	.026	-.145**(-.187)
Grandiose Narcissism × Target Relationship Status × Sex	-.003 (-.003)	.016	.033 (.044)
Grandiose Narcissism × Participant Relationship Status × Sex	.010 (.010)	.186**	.021 (.028)
Target Relationship Status × Participant Relationship Status × Sex	.033 (.033)	.067	.034 (.043)
*R*^2^	.220	.328	.503
Cohen’s *f*^2^	.282	.488	1.012

^#^*p* < .10

*p < .05

**p < .01

***p < .001

Participant Sex was coded -.5 = female, .5 = male; Participant Relationship Status was coded -.5 = single, .5 = attached; Target Relationship Status was coded -.5 = single and .5 = attached. Significant results are emphasized in **bold**.

**Fig 3:**
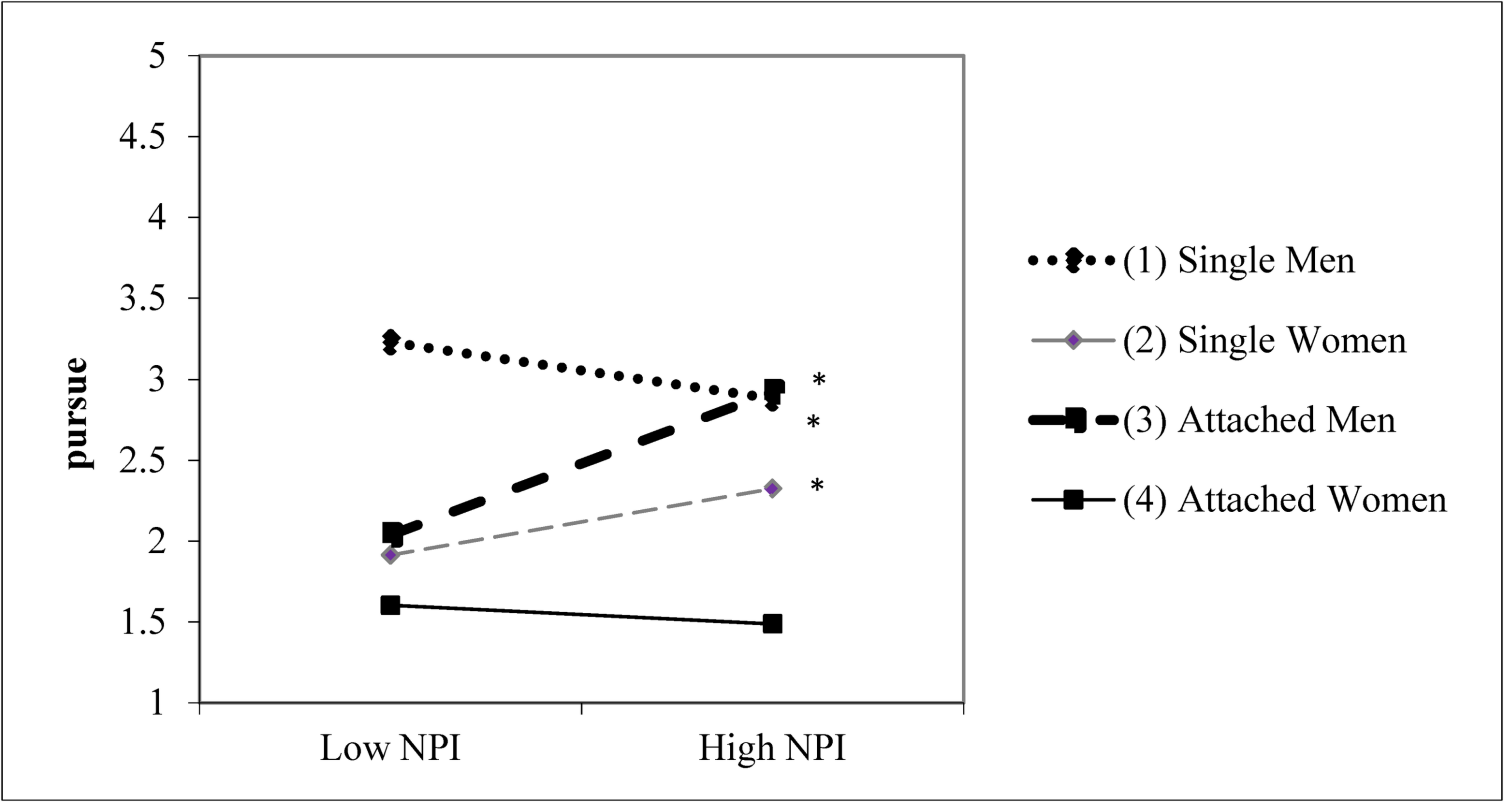
Narcissism × participant relationship status × sex for the likelihood of pursuing the target for a relationship. Asterisks indicate that the simple slopes for single men, single women and attached men are significant. (Study 4).

For pursuing the target for a relationship, men and single participants were more likely to pursue the target for the relationship. There was also a significant target relationship status × sex interaction, which is summarized in Table 10: men were more interested in the single target than women were, but men and women gave more similar ratings for an attached target. The grandiose narcissism × target relationship status interaction was marginally significant (*β* = .107, *t* = 1.805, *p* = .072). There was a significant three-way interaction between grandiose narcissism, participant relationship status, and participant sex for pursuing the target for a relationship, (*β* = .186, *t* = 3.023, *p* = .003). This three-way interaction is displayed in Fig 3. Data are plotted ±1 *SD* from the mean of grandiose narcissism. The simple slope for attached men was significant (simple slope = .08, *t* = 8.08, *p* < .001), revealing that attached men were more likely to pursue the target at higher levels of grandiose narcissism. The simple slope for attached women was not significant (simple slope = -.01, *t* = -0.64, *p* = .53). The simple slope for single men (simple slope = -.02, *t* = -2.29, *p* = .02) and single women (simple slope = .03, *t* = 2.11, *p* = .04) were also significant. Single women were more likely to pursue the target for a relationship at higher levels of grandiose narcissism whereas single men were less likely to pursue the target for a relationship at higher levels of grandiose narcissism. The differences in slopes between single women and attached women was marginally significant (*t* = 1.65, *p* = .10). All other differences among slopes were significant (*t*s ≥ -2.26, *p*s ≤ .03).

**Table 10:** Gender × target relationship status interactions for mate poaching variables (Study 4).

	Pursue for Relationship		Hook Up	
	Men M (SD)	Women M (SD)	Men M (SD)	Women M (SD)
Single Target	3.113 (1.086)	1.710 (0.909)	3.434 (1.083)	1.456 (0.871)
Attached Target	2.654 (1.235)	2.000 (1.017)	2.885 (1.308)	1.729 (1.048)

**Fig 4:**
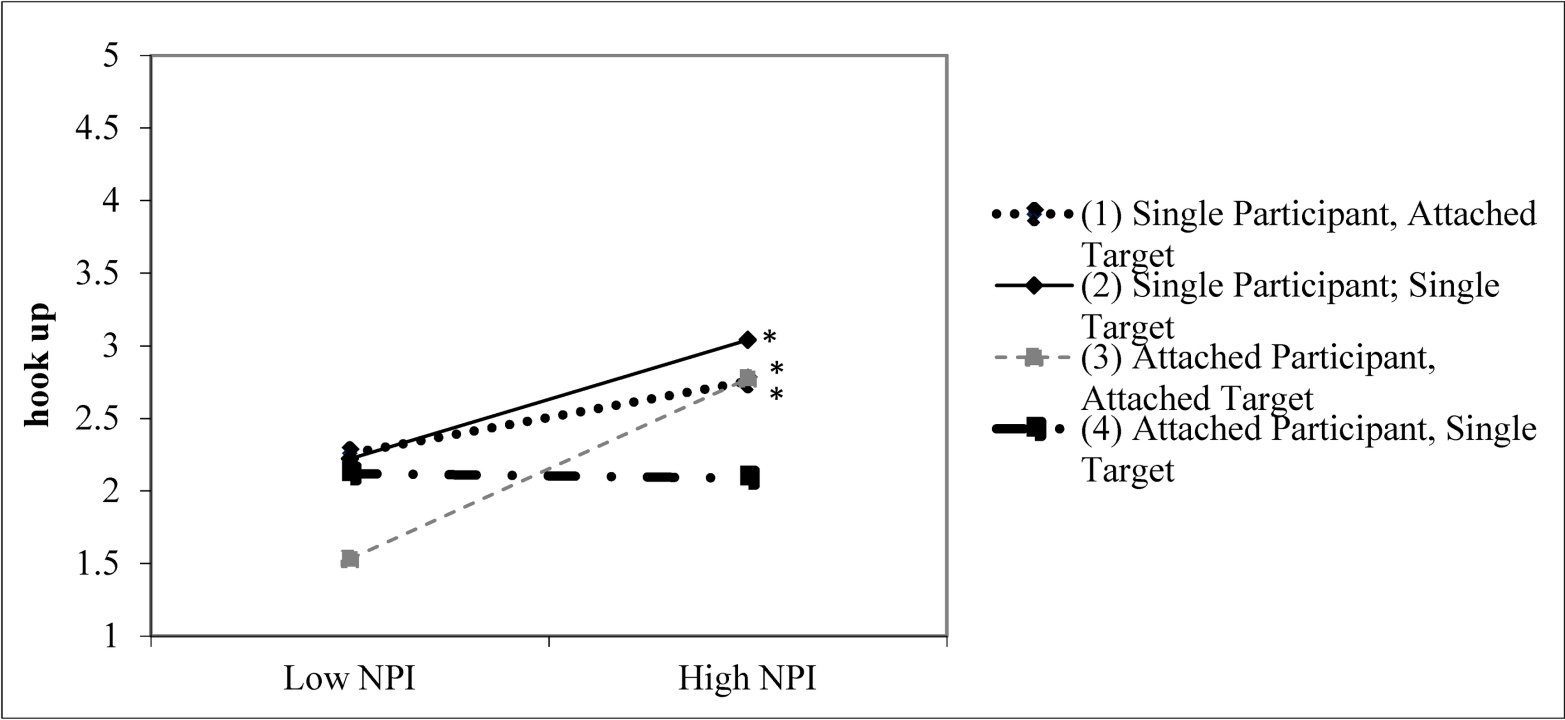
Narcissism × target relationship status × participant relationship status for the likelihood of hooking up with the target. Asterisks indicate that the simple slopes for single participants rating an attached target, single participants rating a single target, and attached participants rating an attached target are significant. (Study 4).

For the likelihood of hooking up with the target, there was a main effect for grandiose narcissism (*β* = .264, *t* = 4.366, *p* < .001), such as that those who were more grandiose narcissistic were more likely to want to hook up with the target. Men and single participants were also more likely to want to hook up with the target. There was a significant grandiose narcissism × sex interaction (*β* = .137, *t* = 2.593, *p* = .010). Grandiose narcissistic men were more likely to want to hook up with the target (simple slope = .08, *t* = 4.51, *p* < .001). The slope for women was not significant (simple slope = .02, *t* = 1.44, *p* = .15). There was also a target relationship Status × sex interaction for hooking up (See Table 10); men were more interested in the single target than women were, but there was less discrepancy in ratings between men and women for an attached target. There was also a grandiose narcissism × participant relationship status × target relationship status interaction (*β* = .145, *t* = 2.774, *p* = .006), which is displayed in Fig 4. The simple slope for a single participant rating an attached target was significant (simple slope = .04, *t* = 3.53, *p* = .001), as were the simple slopes for single participants rating a single target (simple slope = .06, *t* = 8.10, *p* < .001) and attached participants rating an attached target (simple slope = .10, *t* = 7.06, *p* < .001). Only the simple slope for attached participant rating a single target was non-significant (simple slope = -.01, *t* = -0.52, *p* = .60). The slope differences between single participants rating an attached target and single participants rating a single target was not significant (*t* = -.0.96, *p* = .34). All other slope differences were statistically significant (*t*s ≥ -2.50, *p*s ≤ .01).

Last, we assessed the extent to which participants filled out a raffle ticket to get a chance of winning a free date. Thirty-eight participants (16.31%) entered the raffle. Despite this low response rate, we sought to examine if predictor variables would be associated with completing the raffle. We ran logistic regression on all predictor variables to determine whether or not a participant completed a raffle ticket (coded 0 for no and 1 for yes). Our analysis revealed a significant main effect for grandiose narcissism (*B* = .10, *S*.*E*. = .04, *Wald* = 5.86, *p* = .02) and a main effect for the participant’s relationship status (*B* = 1.78, *S*.*E*. = .72, *Wald* = 6.18, *p* = .01). This analysis revealed that more grandiose narcissistic people were more likely to enter the raffle and that attached participants were more likely to enter the raffle. None of the other predictor variables were significant.

## Discussion

In summary, for the likelihood of pursuing a relationship, single men who were lower on grandiose narcissism indicated the greatest interest in pursuing a target for a relationship and single men’s interest in pursuing the target for a relationship declined at higher levels of grandiose narcissism. This pattern likely reflects grandiose narcissists’ lack of interest in pursuing long-term committed relationships [14, 15]. However, attached men’s interest in pursuing the target for a relationship increased at higher levels of grandiose narcissism, which highlights attached grandiose narcissistic men’s prowess and propensity to cheat [8, 12, 13, 38] rather than a preference for a target who is already in a relationship (i.e., to mate poach). Single women were more interested in the target when they were more grandiose narcissistic, but grandiose narcissism did not appear to factor into attached women’s likelihood of pursuing the target for a relationship.

For the item assessing the likelihood of hooking up, a different pattern emerged. Single grandiose narcissists were more interested in pursuing the single target, but attached grandiose narcissists were more interested in a target in a relationship. Pursuing a target who is single is the path of least resistance, but attached grandiose narcissists might be more interested in the attached target because there is less cost of getting caught for having a hook up (the target would not be motivated to tell if he/she was also in a relationship) and also because the target would not be interested in pursuing a long-term relationship with the grandiose narcissist, which is a fit for grandiose narcissists, given their preference for short-term affairs [14]. Single people who were low in grandiose narcissism did not differ in their preference for a short-term relationship with a target who was in a relationship to someone who was single.

## General discussion

Consistent with other self-report research [17, 18], our Studies 1 and 2 found that grandiose narcissists report that they have more frequently engaged in mate poaching. However, Studies 3 and 4 suggest that grandiose narcissists are not more interested in targets who are already in a relationship. Meta-analytically combined results from Studies 3 and 4 are also presented in Table 11; the increased power from combining results demonstrate that grandiose narcissists are not more attracted to or interested in pursuing a person who is already in a relationship. Taken together, the results suggest that grandiose narcissists do not factor in the relationship status of the target when determining their interest in somebody. The only exception was when there appeared to be a low cost for a sexual encounter, such as hooking up with someone who is already in a relationship. Given that grandiose narcissists prefer short-term sexual encounters, there appears to be low risk of entanglement when being asked about hooking up with someone who is already in a relationship because there is less danger that this hook up would turn into a long-term relationship.

**Table 11:** Regression analyses predicting mate poaching variables (combined).

	Physical Attractiveness β (*pr*)	Long-Term β (*pr*)	Short-Term β (*pr*)
Grandiose Narcissism	-.005 (-.004)	.050 (.047)	.151***(.150)
Extraversion	.065 (.060)	.084^#^ (.080)	.085^#^ (.086)
Agreeableness	.033 (.030)	.089^#^ (.086)	.028 (.023)
Conscientiousness	.021 (.021)	-.030 (-.030)	-.048 (-.042)
Neuroticism	.026 (.022)	.027 (.024)	.031 (.023)
Openness	-.059 (-.064)	-.069^#^ (-.077)	-.083*(-.079)
Participant Sex (-.5 = female, .5 = male)	.450***(.406)	.270***(.267)	.412***(.361)
Participant Relationship Status (-.5 = Single, .5 = Attached)	.062 (.066)	.368***(.376)	.283***(.316)
Target Relationship Status (-.5 = Single, .5 = Attached)	-.025 (-.026)	-.033 (-.036)	-.032 (-.038)
Grandiose Narcissism × Target Relationship Status	-.002 (-.002)	.070^#^ (.078)	.071^#^ (.086)
Grandiose Narcissism × Sex	.017 (.019)	.061 (.068)	.132***(.155)
Grandiose Narcissism × Participant Relationship Status	-.046 (.-.048)	-.047 (-.051)	-.001 (-.001)
Target Relationship Status × Sex	-.019 (-.020)	-.102*(-.112)	-.068^#^ (-.079)
Target Relationship Status × Participant Relationship Status	.023 (.025)	-.009 (-.010)	-.009 (-.010)
Sex × Participant Relationship Status	.011 (.012)	.023 (.026)	.057 (.068)
Grandiose Narcissism × Target Relationship Status × Participant Relationship Status	-.005 (-.005)	-.035 (-.039)	-.082*(-.097)
Narcissism × Target Relationship Status × Sex	-.002 (-.002)	.016 (.018)	.028 (.033)
Narcissism × Participant Relationship Status × Sex	.024 (.025)	-.178***(-.191)	-.067^#^ (.-.078)
Target Relationship Status × Participant Relationship Status × Sex	-.030 (-.031)	-.022 (-.024)	.018 (.021)
*R*^2^	.213	.356	.248
Cohen’s *f*^2^	.271	.553	.330

^#^*p* < .10

*p < .05

***p < .001

Participant Sex was coded -.5 = female, .5 = male; Participant Relationship Status was coded -.5 = single, .5 = attached; Target Relationship Status was coded -.5 = single and .5 = attached.

An interesting but unexpected pattern of findings emerged for attached women versus attached men in Studies 3 and 4. Attached men indicated they were more interested in pursuing the target when they scored higher in grandiose narcissism. This pattern is consistent with previous research on grandiose narcissism and romantic relationships, which shows that grandiose narcissists cheat and play games [8, 12, 13, 38]. Attached women, on the other hand, indicated they were less interested in pursuing the target when they scored higher on grandiose narcissism. It could be that when grandiose narcissistic women decide to be in a committed relationship with someone, they feel this person is “the best” potential romantic partner and others pale in comparison. Future research is needed to further examine grandiose narcissistic women’s behavior in relationships.

### Strengths, limitations, and future directions

A strength of the present research is that we were able to go beyond cross-sectional questionnaire approaches to examine grandiose narcissists’ behavior. The paradigm we used was able to determine if grandiose narcissists are more interested in pursuing sexual partners who are already in established relationships. Given the growing number of websites and apps designed to help people “hook up” or date, we believed that this paradigm was realistic. However, we note that our participants were using our “dating website” as part of our study design; these people may or may not use them in their daily lives, and perhaps are even less likely to do so when they are in an ongoing relationship. Additionally, although we used an established paradigm [36], participants only saw a single target. Increasing the number of targets in the paradigm would increase the reliability of the results. Thus, we remain limited in our understanding of how people behave in “the real world” where many factors may be operating, including receiving signals from others that they are interested in having a sexual relationship. One potential way of examining this question is to use an interaction diary that enables the researcher to examine both the participant’s interest in a target as well as the participant’s perception that the target is interested in him/her as well.

Another limitation of our research is that it was focused on responses from predominantly White college student samples. It is possible that responding would be different among a more diverse group, such as contemporaries who are not in college, non-white people, older individuals, and married individuals. Additionally, in Studies 3 and 4 we focused on heterosexual relationships. This occurred because we were limited in our ability to “rig” the computer program to account for both the participant’s sex and the participant’s sexual orientation. However, it is possible that non-heterosexual sexual relationships operate differently than heterosexual sexual relationships. For example, gay men tend to indicate that it is less important to be monogamous than do straight men, straight women, and lesbian women [39]. Therefore, it is possible that the pattern of results might differ by sex and sexual orientation. Future research is needed to examine this question.

In addition, there are three paths for future research to examine. First, the present paper focused on grandiose narcissism, but more research on vulnerable narcissism and relationship dynamics is also needed. A second avenue for future research is to examine why people mate poach. To the best of our knowledge, nobody has yet to examine this question, but research of this kind could potentially be theoretically meaningful. Lastly, future research could examine whether correlations exist between reports of mate poaching and behavioral intentions to mate poach in paradigms such as the one we used in Studies 3 and 4, which would further clarify the relationship between mate poaching intention and actual behavior.

### Conclusion

Although grandiose narcissists tend to report having engaged in more frequent mate poaching tendencies, they do not report a greater interest in people who are already partnered. It appears that grandiose narcissists pursue who they are most interested in rather than taking the other’s relationship status into consideration.
